# The Prevalence of Depression in White-European and South-Asian People with Impaired Glucose Regulation and Screen-Detected Type 2 Diabetes Mellitus

**DOI:** 10.1371/journal.pone.0007755

**Published:** 2009-11-09

**Authors:** Navneet Aujla, Keith R. Abrams, Melanie J. Davies, Nick Taub, Timothy C. Skinner, Kamlesh Khunti

**Affiliations:** 1 Department of Health Sciences, University of Leicester, Leicester, United Kingdom; 2 Department of Cardiovascular Sciences, University of Leicester, Leicester, United Kingdom; 3 Combined Universities Centre for Rural Health, Geraldton, Australia; University of Muenster, Germany

## Abstract

**Background:**

There is a clear relationship between depression and diabetes. However, the directionality of the relationship remains unclear and very little research has considered a multi-ethnic population. The aim of this study was to determine the prevalence of depression in a White-European (WE) and South-Asian (SA) population attending a community diabetes screening programme, and to explore the association of depression with screen-detected Type 2 diabetes mellitus (T2DM) and impaired glucose regulation (IGR).

**Methodology/Principal Findings:**

Participants were recruited from general practices in Leicestershire (United Kingdom) between August 2004 and December 2007. 4682 WE (40–75 years) and 1327 SA participants (25–75 years) underwent an Oral Glucose Tolerance Test, detailed history, anthropometric measurements and completed the World Health Organisation-Five (WHO-5) Wellbeing Index. Depression was defined by a WHO-5 wellbeing score ≤13. Unadjusted prevalence of depression for people in the total sample with T2DM and IGR was 21.3% (21.6% in WE, 20.6% in SA, p = 0.75) and 26.0% (25.3% in WE, 28.9% in SA, p = 0.65) respectively. For people with normal glucose tolerance, the prevalence was 25.1% (24.9% in WE, 26.4% in SA, p = 0.86). Age-adjusted prevalences were higher for females than males. Odds ratios adjusted for age, gender, and ethnicity, showed no significant increase in prevalent depression for people with T2DM (OR = 0.95, 95%CI 0.62 to 1.45) or IGR (OR = 1.17, 95%CI 0.96 to1.42).

**Conclusions:**

Prior to the knowledge of diagnosis, depression was not significantly more prevalent in people with screen detected T2DM or IGR. Differences in prevalent depression between WE and SA people were also not identified. In this multi-ethnic population, female gender was significantly associated with depression.

## Introduction

Psychological distress and depression can often manifest in people with chronic illnesses [1]. Previous research has suggested the presence of an association between depression and Type 2 diabetes mellitus (T2DM) [2]–[13]. A recent meta-analysis found that the odds of co-morbid depression were greater amongst people with diabetes compared to those without [1]. Ethnic disparities were also found for prevalent co-morbid depression in individuals with T2DM, whereby significantly higher rates were identified in South-Asian compared to White-European people, and adjustments for age and gender did not alter these differences [14]. Additionally, other research has argued that depression may be a consequence of living with a diagnosis of diabetes [6], particularly considering the psychological issues relating to disease acceptance and self-management, and the health concerns that may arise from the experience of complications [8]. A study by Knol et al [9] for example, found that the incidence of anti-depressant use was highest 2 months prior to and 3 months following initiation of treatment for T2DM, thereby suggesting the potential impact of diagnosis, commencement of a medication schedule, and disease burden on the psychological wellbeing of patients newly identified with the condition. Interestingly, however, the Diabetes and Self-Management for Ongoing and Newly Diagnosed Diabetes (DESMOND) trial, found comparable rates of depression in people newly diagnosed with T2DM and the general population [15].

It is apparent, therefore, that the underlying mechanism of the relationship between depression and diabetes still remains unclear [6]. What is evident, however, is that the resulting physical, psychological, social and economic implications are dire [4]. People with depression and diabetes often experience poorer quality of life, self-neglect in terms of diet, physical activity and treatment adherence, and increased morbidity and mortality [1]. Consequently, it is important to improve the understanding of this association to further guide development and implementation of preventative strategies, and therefore improve this morbidity [6].

At present, very little research has considered the relationship between depression and previously unrecognised glucose intolerance or screen-detected diabetes. In contrast to people with diabetes and co-morbid depression, individuals with screen-detected diabetes and depression would be unaware of their glucose tolerance status prior to undergoing an Oral Glucose Tolerance Test (OGTT). It is, therefore, important to explore the relationship between screen-detected diabetes and depression further, particularly given the potential, through longitudinal studies, to investigate the directionality and time-line associated with the experience of depression.

Recent cross-sectional studies with a screen-detected population completed in Germany [6] and the US [8] did not find an association between depressive symptoms and unrecognised glucose intolerance, though the latter study found significantly lower prevalence of depressive symptoms in males relative to females. A recent study from the UK [16] found the odds of diabetes to be significantly greater in people with diabetes, in addition to a significant association of 2 hour plasma glucose following an OGTT with depression scores on the Hospital Anxiety and Depression Scale (HADS). The population under investigation in this study was, however, comprised of a high proportion of people from more affluent socio-economic backgrounds and people from an older age group (mean age of 66 years). Further, the ethnicity of study participants was not considered.

Although these studies seemed to indicate that the experience of depression may occur long after a diagnosis of T2DM, the results are inconclusive and require further investigation [6], [8]. Very little research has been completed in the UK for a screen-detected or ‘at risk’ population, and specific consideration has not been made to ethnicity related disparities. South-Asian people are recognised, however, as having an increased susceptibility for the development of T2DM [17], [18] and also for depression [19]. Asghar et al [20] found high overall rates of depression in native South-Asian people with newly recognised diabetes, which were comparable to those in the general population of Bangladesh. The study also found that prevalent depression was again consistently higher amongst females compared to males.

The present study is aiming to investigate the prevalence of depression in South-Asian and White-European people attending a diabetes screening programme. The study also proposes to further examine the association of depression with screen-detected diabetes and impaired glucose regulation (IGR).

## Methods

### Participant Recruitment

The study was carried out in accordance with the principles summarised in the 1996 Helsinki Declaration, and all participants provided written informed consent of their willingness to be involved. After obtaining ethical approval from the Leicestershire local research ethics committees and the Leicestershire, Northamptonshire and Rutland Research Committee 1, participants were recruited to this community screening study.

Participants were recruited from 46 general practices in Leicestershire (UK) between August 2004 and December 2007. Baseline measurements were taken during this time period. A member of the research team met with participating practices to review patient lists and provide assistance with the process of identifying patients eligible to participate, as well as with extraction of patient data. For maintenance of confidentiality, all initial database searches and mailings were performed by practice staff. White-European individuals aged between 40 and 75 were recruited to participate in this study, while people of South-Asian ethnicity were recruited from the age of 25 to 75 years. South-Asian people were identified using the Nam Pehchan software, developed by Bradford City Council from census data [21]. People were only eligible to participate provided that they had not previously received a diagnosis of T2DM. Though, many of the participants had at least one risk factor for T2DM present at the time of recruitment. People with a terminal illness were excluded.

All eligible patients (65,416 people) were posted details about the study, together with a returnable request form for language-specific information written in the major South-Asian languages (Hindi, Gujarati, Bengali, Urdu and Punjabi). Patients expressing an interest in the study were offered individual screening appointments (32, 296 people). Screening took place at the two main hospitals in Leicester (UK), in general practices or on a purpose built mobile screening unit located in their local community, by qualified research nurses. Those patients who did not respond to the first mailing were sent a further mailing within 6 months. Overall, 6749 people were screened which demonstrates a low uptake of screening, with only around 20% of those invited attending for screening. The present paper involves a sub-set of this cohort.

### Assessments

Glucose tolerance was assessed using a 75 g OGTT, whereby individuals were asked to fast for eight hours prior to attending a screening appointment and also to bring a list of prescribed medications with them. Participants were classified by the World Health Organisation (WHO) diagnostic criteria [22]. IGR was defined as Impaired Fasting Glycaemia (IFG) (fasting glucose ≥6.1 and <7.0 mmol/l) and/or Impaired Glucose Tolerance (IGT) (2 hour glucose ≥7.8 and <11.1 mmol/l). Cut-off points for diabetes were fasting glucose ≥7.0 and/or 2 hour glucose ≥11.1 mmol/l [22].

Depression was assessed with the World Health Organisation-Five Wellbeing Index (WHO-5), which is designed to assess the absence of positive mood, using five-items on a six-point frequency scale [23]. WHO-5 attempts to establish a score for the extent to which a person has experienced positive well-being in the two weeks prior to assessment [24], [25]. Although raw scores can vary between 0 and 25, lower scores demonstrate a poor overall feeling of well-being [26]. A cut-point of less than or equal to 13 (≤13) has been proven to offer a 94% sensitivity and 65% specificity as a diagnostic tool for depression [26] with acceptable findings for internal consistency (a Cronbach's alpha of 0.83 [27]). Compared with the Brief Patient Health Questionnaire (B-PHQ) and General Health Questionnaire (GHQ), the WHO-5 was found to be superior for use as a screening tool for depression in primary care [26]. For this study, the English language version of the WHO-5 was used for all participants. Translated versions of the WHO-5 are not currently available for the main South-Asian languages. However, the scale has been used in a native Indian population [28].

Participants also completed a detailed history, including; family history of T2DM, smoking status, and physical activity levels, by self-report. A diagnosis of diabetes was assessed with computer searches of general practice medical records, and data on other medical conditions (including, pre-existing history of depression) was self-reported by patients on examination. Physical activity was measured with the short-format International Physical Activity Questionnaire (IPAQ), which assesses moderate to vigorous intensity activities carried out for greater than 10 minutes within the previous 7 days, using four generic items [29]. Anthropometric measurements, such as; height, weight, and waist circumference were also obtained. Further biochemical measurements, including; glycosylated haemoglobin (HbA1c) were also assessed. Demographic details were also obtained from participants; their postcode was used to calculate the Index of Multiple Deprivation (IMD), which is a deprivation score for small areas in England based on a combination of a number of domains encompassing economic, social and housing factors, and enables ranking of areas according to their specific level of deprivation [30].

### Statistical Methods

The present analysis included only those individuals involved in the study who were of White-European or South-Asian ethnicity. Additionally, as South-Asian participants were recruited from a younger age than White-Europeans, the analysis focused only on those individuals within the 40 to 75 year age group. Participants with missing data were excluded prior to statistical analysis. Throughout the present analysis, statistical significance was assessed at the 5% level.

Unadjusted prevalence figures for depression (with 95% confidence intervals) were calculated for T2DM, IGR and people with normal glucose tolerance (NGT), separately for White-European and South-Asian participants, and males and females. Interactions between ethnicity and T2DM, IGR and NGT were investigated, as were interactions of sex with T2DM, IGR and NGT. Age-stratified prevalence figures were calculated following categorisation of the continuous age variable into two strata (40–59, 60 and over). The age-adjusted prevalence was calculated based on a logistic regression model. Continuous age was used to calculate the age-adjusted prevalence.

In addition, continuous relationships between wellbeing score and fasting and two-hour plasma glucose levels were explored with scatterplots. A logarithmic transformation to base e was attempted for the highly skewed plasma glucose measurements.

To analyse the association of depression with screen-detected T2DM and IGR, logistic regression was performed with depression (yes/no) as the outcome, and T2DM and IGR as predictor variables. Initially, an investigation into the potential confounders of this relationship was undertaken using the t-test for continuous variables and the chi-squared test for categorical data. The Bonferroni adjustment was also applied to account for multiple testing. Variables related to both T2DM or IGR and depression were included in a logistic regression model. Age, gender, and ethnicity were forced into the logistic regression model regardless of statistical significance, on the basis that these variables were important effect modifiers rather than confounding factors. Odds ratios and 95% confidence intervals (95%CI) were obtained. Statistical analyses were completed using STATA 9 (StataCorp LP, College Station, TX, USA).

## Results

### Descriptive Analysis

Descriptive statistics are summarised in table 1. Overall, 6009 participants were involved in the analysis of this study; 4682 (78%) people of White-European (WE) ethnicity, and 1327 (22%) South-Asians (SA). Table 1 shows that the unadjusted prevalence for screen-detected T2DM was 3.4% (95%CI 2.9–3.8) and 14.4% (95%CI 13.4–15.1) for IGR.

**Table 1:** Descriptive statistics for the study population, mean (standard deviation) unless otherwise stated.

Gender (N (%))		
Male	2849 (47)	
Female	3160 (53)	
Ethnicity (N (%))		
White-European	4682 (78)	
South-Asian	1327 (22)	
Diagnosis (N (%))		
Type 2 diabetes	198 (3.4)	
Impaired glucose regulation	855 (14.4)	
Normal	4956 (82.5)	
Depressed (N (%))	1231 (25.2)	
Age (years)	N = 6003	58(9.7)
Fasting glucose at screening (mmol/l)	N = 6001	5.2 (0.9)
2-hour glucose at screening (mmol/l)	N = 5973	6.0 (2.5)
Hba1C (%)	N = 5941	5.7 (0.6)
Wellbeing Score	N = 4895	16 (5)
Body Mass Index (BMI) (kg/m^2^)	N = 5980	28.1 (5)
Waist (cm)	N = 5977	94.2 (13.2)
Index of Multiple Deprivation (IMD) Score	N = 5337	18.2 (12.3)
Smoking status (N (%))		
Non-smoker	3425 (57)	
Current smoker	847 (14)	
Ex-smoker	1713 (28.5)	
Total	5985	
Physical activity (N (%))		
Low	1434 (25.9)	
Moderate	939 (17)	
Vigorous	3163 (57.1)	
Total	5536	
Family history of Type 2 diabetes (N (%))		
First degree relative	1338 (22.3)	
Non-first degree relative	122 (2)	
None	4547 (75.7)	
Total	6007	

Table 2 illustrates that South-Asian participants were significantly younger than the White-Europeans. Fasting and 2-hour plasma glucose levels were significantly higher amongst South-Asian participants, as was HbA1c. South-Asian people had significantly lower body mass index (BMI) than White-Europeans, as well as a significantly lower waist circumference. South-Asian people also had a significantly higher IMD score, which indicated a greater level of deprivation than White-European participants. South-Asian people were significantly less often a current smoker or an ex-smoker, were less often physically active and more often had a family history of T2DM, compared to White-European participants.

**Table 2:** Differences in characteristics between White-European and South-Asian participants, mean (standard deviation) unless otherwise stated.

	White-European (N = 4688)		South-Asian (N = 1340)		P-Value
Age (years)	N = 4678	59.2 (9.6)	N = 1325	53.6 (8.8)	<0.001
Wellbeing Score	N = 4131	16.15 (4.71)	N = 764	16.16 (5.07)	0.96
Fasting glucose at screening (mmol/l)	N = 4677	5.2 (0.9)	N = 1324	5.3 (0.9)	0.001
2-hour glucose at screening (mmol/l)	N = 4654	5.9 (2.4)	N = 1319	6.5 (2.7)	<0.001
Hba1C (%)	N = 4641	5.7 (0.6)	N = 1300	5.9 (0.6)	<0.001
Body Mass Index (BMI) (kg/m^2^)	N = 4655	28.3 (5)	N = 1325	27.5 (4.9)	<0.001
Waist (cm)	N = 4655	94.7 (13.5)	N = 1322	92.6 (11.9)	<0.001
Index of Multiple Deprivation (IMD) Score	N = 4225	16.9 (11.7)	N = 1112	23.5 (13.1)	<0.001
Depressed status (Yes (%)/Total)	1028 (24.9)/4131		203 (26.5)/764		0.32
Smoking status (N (%))					
Non-smoker	2312 (49.6)		1113 (84.3)		<0.001
Current smoker	743 (15.9)		104 (7.9)		<0.001
Ex-smoker	1610 (34.5)		103 (7.8)		<0.001
Total	4665		1320		
Physical activity (N (%))					
Low	925 (20.9)		509 (46)		
Moderate	765 (17.3)		174 (15.7)		
Vigorous	2740 (61.9)		423 (38.2)		
Total	4430		1106		<0.001
Family history of Type 2 diabetes (N (%))					
First degree relative	3655 (78)		892 (67.3)		
Non-first degree relative	102 (2.2)		20 (1.5)		
None	924 (19.7)		414 (31.2)		
Total	4687		1326		<0.001

81% of participants had complete data for the WHO-5. Though significant differences in wellbeing scores could not be established between White-European and South-Asian participants, wellbeing scores were found to be significantly lower among female participants than males (mean = 16 (SD = 4.92) versus 17 (SD = 4.53) in females and males respectively, p<0.001).

Further descriptive analyses were not able to identify a relationship of wellbeing score with logarithmic transformed fasting and two-hour plasma glucose levels for White-European or South-Asian participants. Also, 1.7% of participants were found to have a pre-existing history of depression with varying levels of severity (including, mixed depression and anxiety, bipolar disorder and co-morbid depression). Of this proportion, 3.9% had screen-detected T2DM and 16.3% had IGR.

### Prevalence of Depression

Unadjusted prevalence figures are presented in table 3. Prevalence figures were very similar for T2DM, IGR and NGT, though were slightly elevated for the latter two groups compared to T2DM. Statistical significance in the differences between prevalent depression between males and females, or White-European and South-Asian people could not be established. However, prevalence figures were elevated for females compared to males with T2DM, IGR or NGT.

**Table 3:** Unadjusted prevalence of depression for White-European and South-Asians, and males and females.

	T2DM	IGR	NGT
	% Prevalence (95% CI)		
	r/n		
White-Europeans	21.6 (14.0–30.8)	25.3 (21.7–29.3)	24.9 (23.5–26.4)
	22/102	134/529	872/3500
South-Asians	20.6 (8.7–37.9)	28.9 (20.8–38.2)	26.4 (23.0–30.1)
	7/34	33/114	163/616
P-value	0.75	0.65	0.86
Males	18.4 (10.8–28.1)	19.9 (15.7–24.8)	21.1 (19.3–23.0)
	16/87	63/316	412/1952
Females	26.5 (14.9–40.1)	31.8 (26.8–37.2)	28.8 (26.9–30.7)
	13/49	104/327	623/2164
P-value	0.95	0.29	0.27
Overall	21.3 (14.8–29.2)	26.0 (22.6–29.5)	25.1 (23.8–26.5)
	29/136	167/643	1035/4116

r denotes observed number with depression in each group.

n denotes total number of people in that particular group.

Age-adjusted prevalence figures (table 4) were consistently increased for females compared to males, particularly amongst women of South-Asian ethnicity. Though, prevalent depression did not differ significantly by sex and ethnicity (p = 0.38). Across all groups, prevalence figures were greatest for those with IGR, and were lower among those with screen-detected T2DM; however, these differences were only marginal. It was clear, however, that depression was more prevalent in people with NGT, in comparison to those with T2DM. Overall, age-adjusted prevalences were elevated across T2DM, IGR and NGT, and there was only marginal variation in figures across all groups.

**Table 4:** Age-stratified and age-adjusted prevalence of depression for White-European and South-Asians.

	White-European Males			White-European Females			South-Asian Males			South-Asian Females		
	T2DM	IGR	NGT	T2DM	IGR	NGT	T2DM	IGR	NGT	T2DM	IGR	NGT
	% Prevalence											
	(r/n)											
40–59	23.1	26.8	24.3	31.7	36.1	33.2	22.5	26.2	23.7	31.0	35.4	32.4
	(5/23)	(24/84)	(205/833)	(1/6)	(35/98)	(332/1004)	(4/19)	(11/41)	(64/248)	(2/10)	(17/38)	(76/262)
60 or over	15.7	18.5	16.6	22.3	26.0	23.5	15.3	18.0	16.2	21.8	25.4	23.0
	(7/41)	(25/169)	(130/801)	(9/32)	(50/178)	(205/862)	(0/4)	(3/22)	(13/70)	(1/1)	(2/13)	(10/36)
Age-adjusted prevalence (95% CI)	18.2 (12.8–28.0)	21.0 (15.4–32.2)	20.3 (12.0–29.1)	24.1 (18.8–33.1)	29.3 (22.0–42.5)	28.4 (18.7–39.0)	19.6 (12.3–25.7)	22.5 (14.9–30.6)	21.2 (12.6–27.6)	27.9 (18.9–36.1)	32.0 (20.9–40.7)	30.0 (18.1–37.2)

r denotes observed number with depression in each group.

n denotes total number of people in that particular group.

### Association of Depression with Diabetes

A statistically significant association of depression with screen-detected T2DM or IGR was not identified in any of the unadjusted or adjusted models (table 5). Addition of further variables in models 2 and 3 did not affect the relationship between depression and T2DM or IGR. Statistically significant associations of ethnicity with prevalent depression were also not identified (models 2 and 3, table 5). Females had a statistically significant odds ratio of 1.55 compared to males when controlling for age and ethnicity (model 2, table 5), which increased greatly with further adjustment for BMI, current smoking status, physical activity, waist circumference and IMD score (model 3, table 5). Therefore, with adjustment, females had a significant 84% increase in the odds of depression, compared to males. A statistically significant association was apparent for depression with age in models 2 and 3 (table 5). Addition of confounding factors in model 3 did not affect the association of depression with ethnicity or age.

**Table 5:** Logistic regression with depression as the outcome, inclusive of unadjusted models and models adjusted for confounding factors.

	Odds Ratio (95% CI)	P-value
Model 1		
Screen-detected T2DM vs. Normal*	0.81 (0.53–1.22)	0.31
IGR vs. Normal*	1.04 (0.86–1.26)	0.65
Model 2		
Screen-detected T2DM vs. Normal*	0.95 (0.62–1.45)	0.82
IGR vs. Normal*	1.17 (0.96–1.42)	0.12
South-Asian vs. White-European*	0.92 (0.77–1.11)	0.40
Female vs. Male*	1.55 (1.36–1.77)	<0.001
Age	0.97 (0.97–0.98)	<0.001
Model 3		
Screen-detected T2DM vs. Normal*	0.74 (0.44–1.23)	0.24
IGR vs. Normal*	1.10 (0.89–1.36)	0.38
South-Asian vs. White-European*	0.92 (0.75–1.14)	0.47
Female vs. Male*	1.83 (1.52–2.20)	<0.001
Age	0.97 (0.96–0.98)	<0.001
Moderate exercise vs. No moderate exercise*	0.71 (0.57–0.88)	<0.01
Vigorous exercise vs. No vigorous exercise*	0.54 (0.45–0.65)	<0.001
Current smoker vs. Non-current smoker*	1.72 (1.42–2.08)	<0.001
Waist circumference	1.02 (1.01–1.03)	<0.01
BMI	0.98 (0.95–1.01)	0.17
IMD Score	1.01 (1.00–1.02)	<0.01

* Denotes reference category.

Of the confounders included in the model, significant independent associations with depression emerged for waist circumference, IMD and BMI (model 3, table 5). Though the associations for these variables were statistically significant, it is important to note that the odds ratios and 95% confidence intervals only suggested a marginal effect, and these variables were included only as confounding factors. Similarly, following adjustment, significant odds ratios were found for current smokers, people undertaking moderate physical activity and for people undertaking vigorous levels of physical activity (model 3, table 5).

## Discussion

The present study aimed to explore the prevalence of depression in South-Asian and White-European people attending a diabetes screening programme, and also to further examine the association of depression with screen-detected diabetes and IGR. With respect to these aims, the results from the study demonstrated that prior to the knowledge of diagnosis, depression was not significantly more prevalent in people with screen-detected T2DM or IGR. Prevalent depression did not differ significantly across White-European and South-Asian participants, though the study did find that depression was significantly higher in prevalence among females than males.

The findings from the present study are largely consistent with those demonstrated in previous research by Icks et al [6] and Rhee et al [8], whereby an association of depression with screen detected T2DM or IGR could not be identified. Although previous studies have found that co-morbid depression is significantly more prevalent in people with diabetes, the results from this study and others using a screen-detected population are important to add to our understanding of the mechanism of the relationship between depression and diabetes. Findings that demonstrate statistical non-significance for this association in a screen-detected population may indicate the likely progression to developing depression following a diagnosis of diabetes. This may possibly be the case when comparing the present results to those found by Ali et al [14] whose work identified rates of depression in White-European and South-Asian people with established T2DM to be at around 22% and 32%, respectively. It could be that people eligible to participate in diabetes screening studies, such as in the present study, may have an increased susceptibility for depression prior to the knowledge of glucose tolerance status. It would be interesting to follow these people up in a prospective study to add further knowledge to the directionality of the relationship between depression and diabetes. Prevalence figures in the Ali et al study [14] were obtained with the HADS. Comparison of the present results to normative data for the WHO-5 in a UK population has not been possible as this data has not yet been published.

Though statistically significant, differences in fasting and two hour glucose levels between White-European and South-Asian people in the present study cannot be considered as clinically relevant, in accordance with the WHO criteria [22]. Similarly, HbA1c levels are slightly below the 6 or greater (≥6) and less than 6.5 (<6.5) criteria for clinical significance in terms of diagnosing diabetes [31]. According to National Institute for Health and Clinical Excellence (NICE) clinical guidance [32], however, average BMI in the present study can be interpreted as indicative of overweight, particularly with the lower values of BMI that are considered clinically relevant for people of South-Asian ethnicity. Similar can be interpreted from the large waist circumference measurements demonstrated in the present study, in accordance with International Diabetes Federation (IDF) guidelines for clinical significance [33].

The present findings did offer support for previous findings from other studies demonstrating a greater prevalence of depression in females compared to males [1], [6], [20]. The extent to which the findings regarding ethnicity are consistent with other literature, however, is challenging to discuss because of the paucity of studies focusing on the relationship between depression and diabetes in a South-Asian population. This is the first study to investigate these differences between South-Asians and White-Europeans. For the present sample, very few differences were found in prevalent depression across White-European and South-Asian people. A potential explanation for this could be related to the tool used to assess depression in the present study. As previously mentioned, the WHO-5 was implemented to assess well-being of study participants, whereby those with a well-being score of 13 or lower were considered to have poor well-being, and were therefore classified as probable cases of depression. Previous research has documented the adequacy of the ≤13 cut-point for detecting depression (for example, Henkel et al [25]). Although previous literature investigated the ≤13 cut-point on a number of patient sub-groups; for example, males and females, and people aged above or below 56 [25], research has not been completed into evaluating the adequacy of the cut-point for detecting depression in a predominantly South-Asian population. Therefore, though the lack of differences found in prevalent depression between South-Asian compared to White-European participants in the present study may well be a true reflection of the sample, it is important to recognise that the results may also be implying that the well-documented ≤13 cut-point on the WHO-5 may not be appropriate enough to detect depression for South-Asian people.

Similarly, it is also important to note that self-reported depression was not confirmed with a diagnosis in accordance with the Diagnostic and Statistical Manual of Mental Disorders (DSM-IV) from administration of a clinical interview with a psychiatrist or psychologist. Depressed cases in the present study were identified purely on the basis of diagnosis of well-being from the WHO-5. Therefore, the figures presented in this paper may be an over or underestimate of the prevalence of depression, and should really be supplemented with a gold-standard approach for the purposes of future research. There is ongoing debate around measures for wellbeing, whereby some researchers argue for the increased sensitivity in assessing the absence of positive mood in people, in comparison to common measures of depression, which tend to focus on the presence of negative mood.

The present analysis did not have access to any data representing the use of anti-depressants in the sample. WHO-5 assesses wellbeing in the two weeks prior to self-report. For participants who have previously taken anti-depressant medication or are currently receiving treatment for depression, their mood state at the time of measurement may be considerably more positive than would have been if they were not receiving medication. Therefore, the prevalence figures for depression in the present study may not be an adequate representation of the sample, and may be an underestimation. Furthermore, as with most studies investigating the relationship of depression with T2DM, the present study was based on cross-sectional data, so it would not be possible to determine any patterns of causation or temporal relationships.

Although the study was limited in some aspects, it also has a number of strengths. Our study was particularly strong in the large sample size that was available for analysis. This provided a greater statistical power to detect an association of depression with screen detected T2DM and IGR, should any have really existed. We also explored potential confounders in the analysis (for example, age, sex and BMI). On the basis that Ali et al [1] recognised that previous research neglected to adequately account for confounders, the present study was positive in that the models developed were appropriately adjusted for the influence of all potential confounders. A major strength of the study was also in the use of an OGTT to assess glucose tolerance. In addition, our study was the first to investigate the association of depression with unrecognised glucose intolerance in a multi-ethnic population with a high proportion of South-Asian participants. This is obviously an area that has been neglected by previous research but ethnicity related disparities are important to investigate further, given the increasing prevalence of T2DM amongst this population.
